# Supplementary figures and images for: BATF relieves hepatic steatosis by inhibiting PD1 and promoting energy metabolism
Source: eLife. 2023 Sep 15;12:RP88521. doi: 10.7554/eLife.88521 (PMC10503959; doi:10.7554/eLife.88521)

## Slide 1
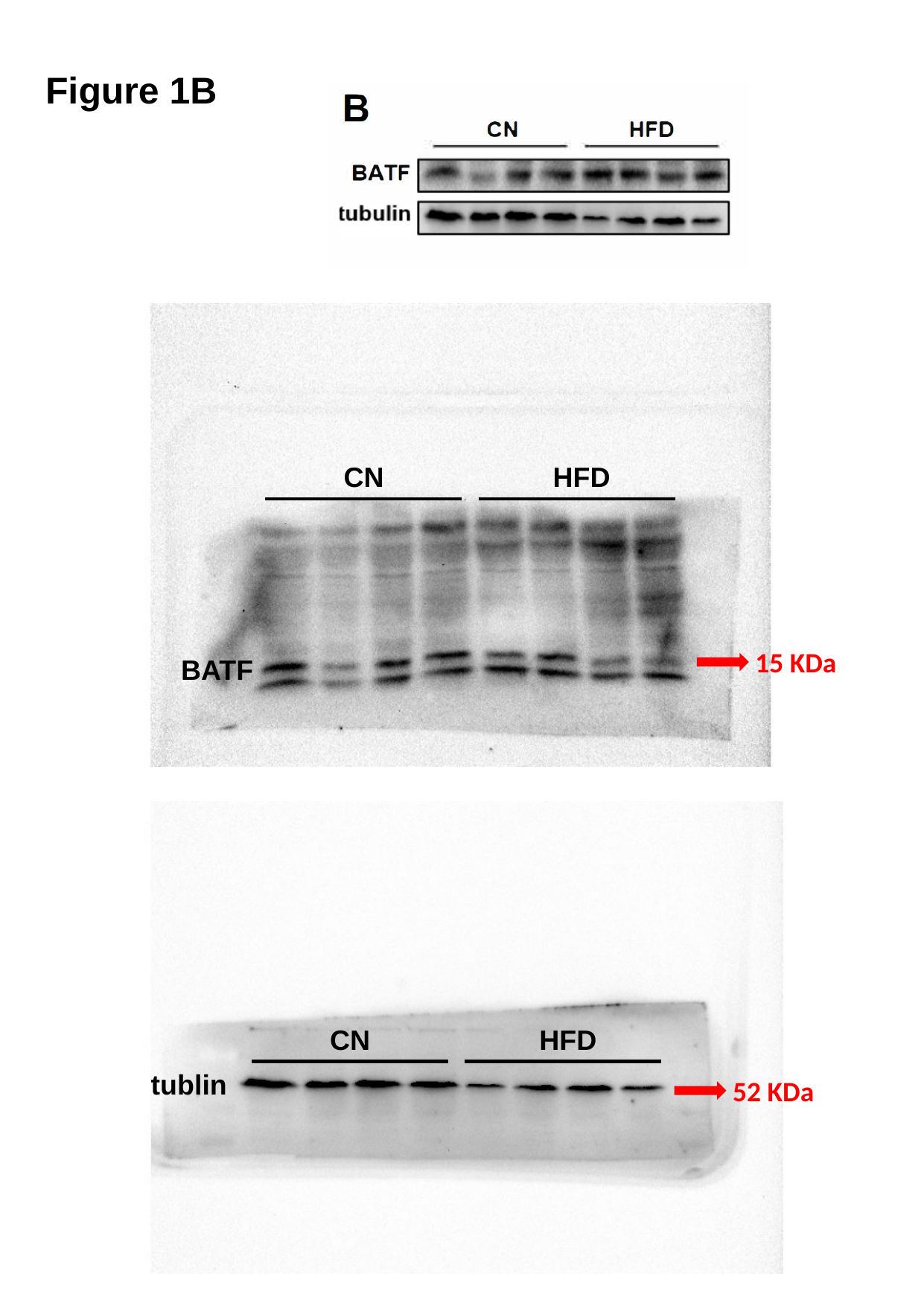

Figure 1B
CN
HFD
15 KDa
BATF
CN
HFD
tublin
52 KDa

## Slide 2
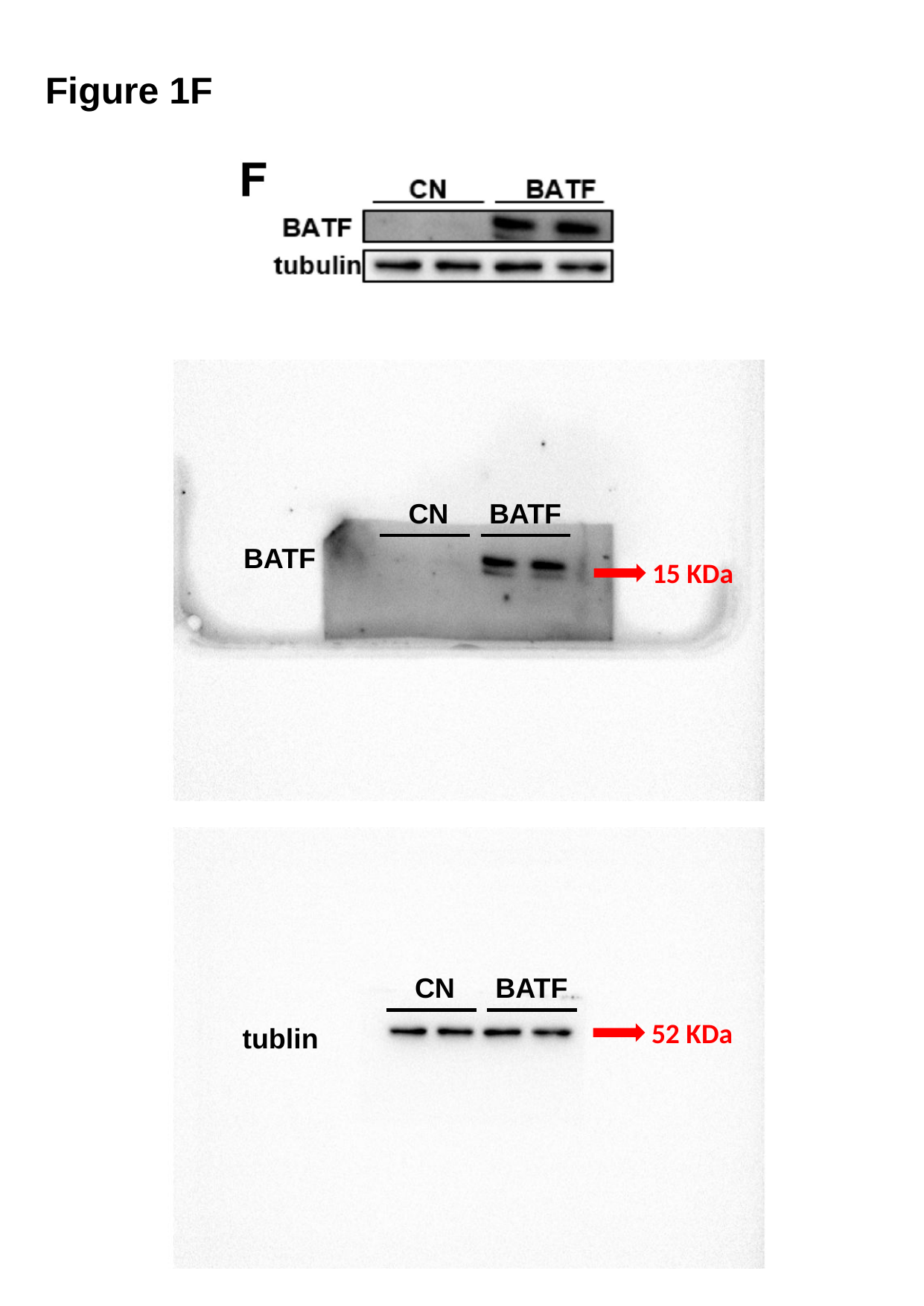

Figure 1F
CN
BATF
BATF
15 KDa
CN
BATF
52 KDa
tublin

Supplement: Figure 1—source data 1. [file elife-88521-fig1-data1.zip › Figure 1-source data 1/Figure 1-Source 2-WB.pptx]
