# Supplementary material for: BATF relieves hepatic steatosis by inhibiting PD1 and promoting energy metabolism
Source: eLife. 2023 Sep 15;12:RP88521. doi: 10.7554/eLife.88521 (PMC10503959; doi:10.7554/eLife.88521)
Supplement: Figure 2—source data 1. [file elife-88521-fig2-data1.zip › Figure 2-source data 1/Figure 2-source data 2-WB.pptx]

## Slide 1
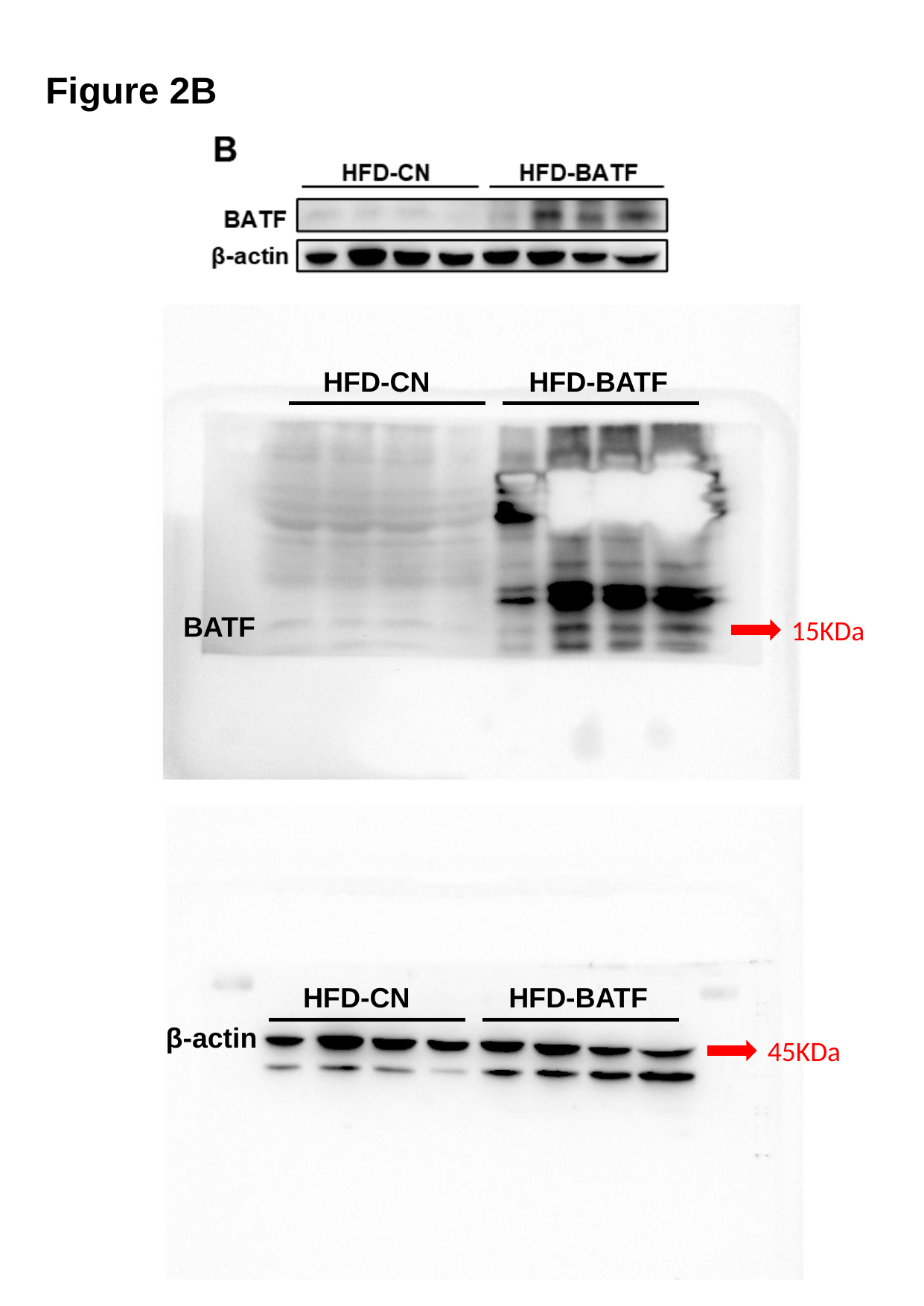

Figure 2B
HFD-CN
HFD-BATF
BATF
15KDa
HFD-CN
HFD-BATF
β-actin
45KDa

## Slide 2
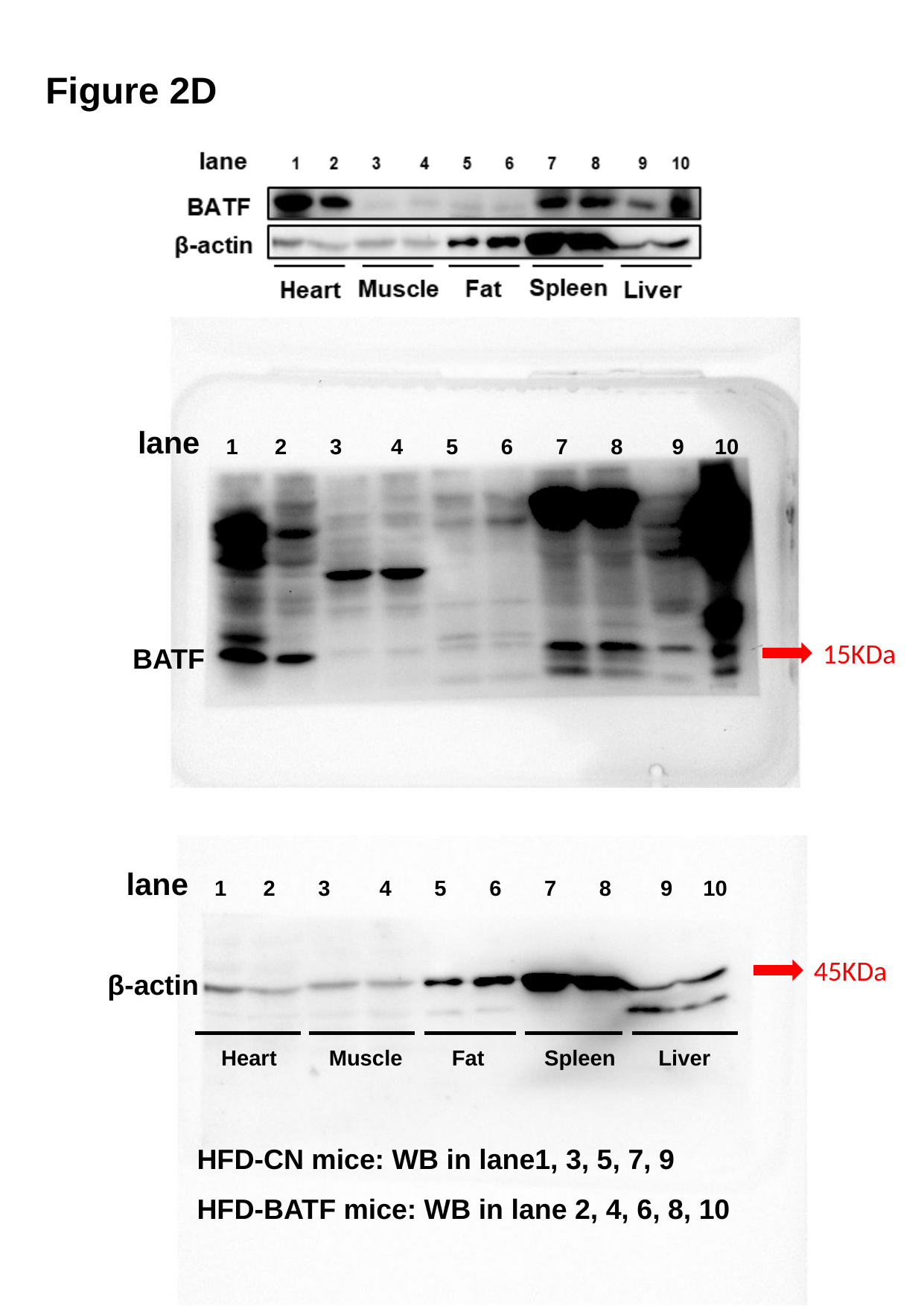

Figure 2D
lane
1 2 3 4 5 6 7 8 9 10
BATF
15KDa
lane
1 2 3 4 5 6 7 8 9 10
β-actin
Heart
Fat
Spleen
Muscle
Liver
HFD-CN mice: WB in lane1, 3, 5, 7, 9
HFD-BATF mice: WB in lane 2, 4, 6, 8, 10
45KDa
